# Mount Vernon Hospital and the Case for Paying Consumptives

**Published:** 1913-03-15

**Authors:** 


### The Hospital

The Workers' Newspaper of Administrative Medicine and Institutional Life, Administration, National Insurance and Health.

No. 1393, Vol. LIII. SATURDAY, MARCH 15, 1913. PRICE ONE PENNY.

### MOUNT VERNON HOSPITAL AND THE CASE FOR PAYING CONSUMPTIVES.

The meeting of the governors of the Mount Vernon Hospital on the 12th instant was held too late in the afternoon to enable us to report the proceedings this week. Inferentially it raises the whole question of the relations and fate of the voluntary chest hospitals in London and indirectly throughout the country. At the annual meeting last year of the governors of the Mount Vernon Hospital the honorary staff opposed power being given to the Committee to separate the two institutions under their control—namely, that at Hampstead and that at Northwood—with a view to the further and better perpetuation of these hospitals. Despite the opposition of the honorary medical staff, the governors at their meeting in March 1912 conferred this power upon the Committee. It is important to keep these points in mind, because it was made plain by the letter of the Secretary of the Local Government Board dated 25th ultimo that the capital grant made available by the Finance Act of 1911 to aid the provision of institutions for the treatment of tuberculosis is a fixed sum, and that early application is essential if the Board is to be placed in a position to make grants of such an amount to a particular institution as the circumstances may justify. The Committee of the Mount Vernon Hospital clearly had this danger in view at the last annual meeting, and had the medical staff then adopted the policy which is now recommended by Dr. Tunnicliffe, the chairman of the medical board of this institution, in his letter to *The Times* of the 7th instant, the present difficulty and dangers which threaten the Mount Vernon Hospital might have been successfully surmounted by this time.

The position of the Mount Vernon Hospital to-day is that eighty of the occupied beds are at present devoted to insured persons, and only thirty are occupied by patients who are not insured. The management of Mount Vernon find that they cannot in present circumstances, we are informed, continue to obtain adequate support from the public in voluntary contributions for the maintenance of this institution, the income showing a diminution during the past twelve months of something like £5,000. The circumstances which affect this class of institution have been entirely changed by the passing of the National Insurance Act, which provides a large sum of money to enable this very work to be done, not only in London but throughout the country, on an adequate and business basis. Such a basis can be properly secured now by negotiations between the chest hospitals, the Local Government Board, the Insurance Commissioners, and the administrative public health authorities of local government in London and throughout the country. Major Cecil B. Levita's letter in *The Times* of the 7th instant and the other correspondence which has appeared there in relation to the Mount Vernon Hospital demonstrate the existence of a desire to bring about a combined effort of all the interests affected to secure that the campaign against tuberculosis in London shall be made thoroughly effective. It would seem, therefore, that the course suggested by Dr. Habershon, as chairman of the medical representatives of the London chest hospitals, in his letter to *The Times* of the 10th instant, is the best, and that all should combine to co-ordinate the different schemes and to work in full co-operation with the hospital authorities.

Reverting to the case of the Mount Vernon Hospital, we may point out that the whole difficulty is at the bottom a financial one. The Committee cannot properly go on struggling with two hospitals, one at Hampstead and another at Northwood, when the funds at their disposal, which are steadily diminishing in amount, already represent a deficiency of one-third of the total annual cost entailed. There can be no doubt of the intention of Parliament by National Insurance to provide an adequate system of chest hospitals for insured persons, including the indigent poor, because the aim of this legislation is to eradicate tuberculosis upon a basis which will render it possible to classify the patients in accordance with the stage of disease from which each may be suffering, and so to attain the maximum of success in treatment. Authorities agree that cases in the early stage must be separated and treated apart from the more advanced cases.

The governors of Mount Vernon Hospital will have the Committee's assurance that they can see their way to maintain and even extend the work of the institution at Northwood, where patients are treated who can afford to pay 25s. a week, and so to defray the cost of their maintenance and treatment. They will further be in a position, if the two institutions are separated and the Committee is freed from the burden of maintaining the Mount Vernon Hospital, to add a pavilion or pavilions at Northwood for the reception of poorer patients, should such a step be found necessary. The governors of Mount Vernon Hospital have to choose between continuing to struggle with a financial burden which must tend to ruin the institution financially, or by leaving the work which under the National Insurance Act properly belongs to the public authorities and confining themselves to their proper field of usefulness which they have successfully initiated at Northwood, to ensure the perpetuation of the objects of the original founders. If they decide wisely the governors may possess an institution of the highest philanthropic value by virtue of the work which it will then do for large classes of the community who do not benefit by National Insurance, and whose circumstances are such as to entitle them to the generous sympathy and support of those who in the past have maintained voluntary institutions with wisdom and energy. Mount Vernon Hospital, if the governors on the 12th instant decide to divorce it entirely from Northwood, and to hand it over to the authorities to be conducted as a chest hospital under their management, may so fulfil to an even greater extent than in the past the objects which its founders had at heart. It is presumed that the authorities will naturally make arrangements for the continuance of Mount Vernon Hospital under the present medical staff, who should thus secure even greater facilities for effective work than they have ever yet enjoyed.

